# Extraordinary effects of unnatural pairings

**DOI:** 10.7554/eLife.27198

**Published:** 2017-05-12

**Authors:** Alejandro Villarino, John J O'Shea

**Affiliations:** National Institute of Arthritis and Musculoskeletal and Skin Diseases, National Institutes of Health, Bethesda, United States; National Institute of Arthritis and Musculoskeletal and Skin Diseases, National Institutes of Health, Bethesda, United StatesJohn.Oshea@nih.gov

**Keywords:** cytokine signaling, cytokine engineering, cytokine therapies, Human

## Abstract

Engineered molecules based on human cytokines have potential uses in research and medicine.

**Related research article** Moraga I, Spangler JB, Mendoza JL, Gakovic M, Wehrman TS, Krutzik P, Garcia KC. 2017. Synthekines are surrogate cytokine and growth factor agonists that compel signaling through non-natural receptor dimers. *eLife* **6**:e22882. doi: 10.7554/eLife.22882

As with John Lennon and Paul McCartney, pairings can sometimes exceed the sum of their parts. Many biological processes also rely on proteins forming pairs: for example, a wide variety of cells (particularly those involved in the immune system) release small proteins called cytokines to communicate with other cells, and typically it takes two cytokine receptors to detect one of these proteins. Now, in eLife, Christopher Garcia and colleagues at Stanford University School of Medicine – including Ignacio Moraga as first author – report that they have developed a new class of molecule called synthekines that bind to different pairs of cytokine receptors than the cytokines they are based on (Moraga et al., 2017).

Each cytokine interacts with a unique cytokine receptor pair and activates signaling pathways that lead to changes in gene expression (Figure 1). In mammals, there are ∼40 cytokine receptors that employ the Jak-STAT signaling pathway: this is an evolutionarily conserved pathway composed of four Jak proteins and seven STAT proteins that cooperate to sense when specific cytokine receptors form pairs (Villarino et al., 2017). Simple math tells us that, if all such receptors could freely associate with each other, there would be 40x40=1,600 combinations of pairs. However, this is not the case; the cytokines present in an organism determine which receptor pairings occur, limiting the actual number of pairings to less than 50.

**Figure 1.**
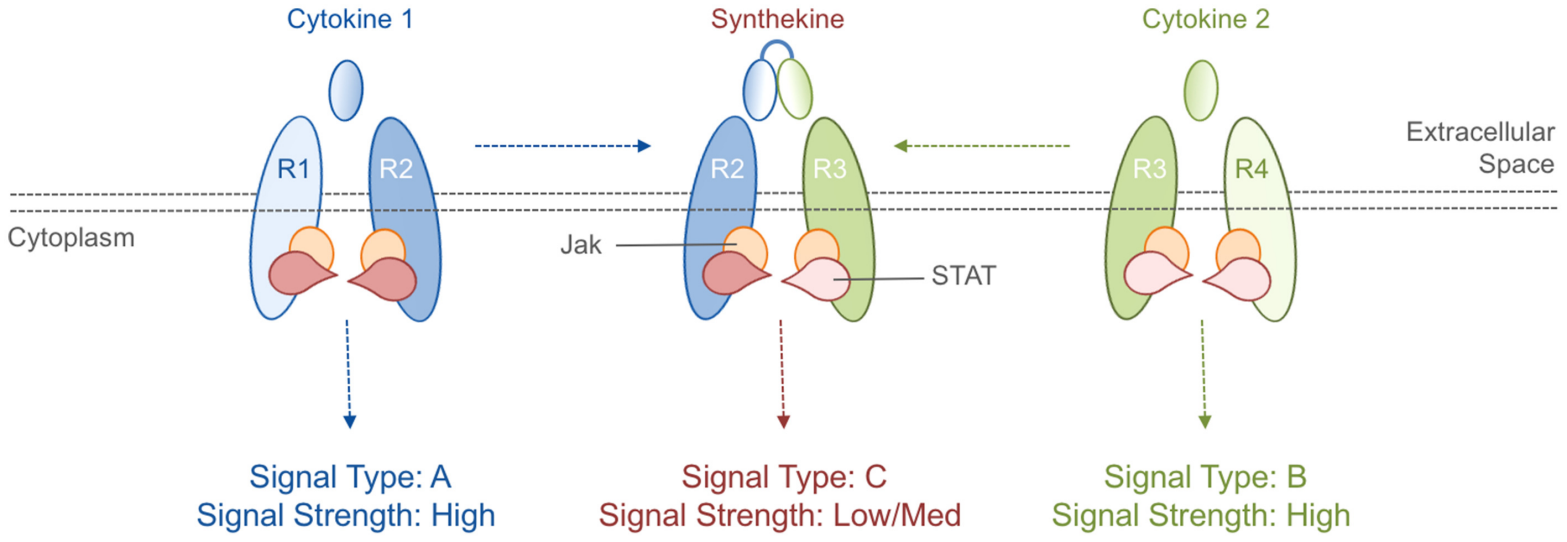
Cytokines and synthekines. The cytokines present in an organism dictate which cytokine receptors can form pairs: in this schematic example, cytokine 1 causes receptors R1 and R2 to form a pair (left), while cytokine 2 causes receptors R3 and R4 to form a pair (right). Moraga et al. made new molecules called synthekines, with each synthekine being a composite of two mutant cytokines that each bind only one of their related receptors. These synthekines trigger the formation of ‘unnatural’ pairs of receptors (R2 and R3 in this case). The Jak-STAT signaling cascade downstream of the synthekine is weaker than the cascades triggered by the parent cytokines: there are also other differences in the signaling cascades (see main text).

Cytokines bind to a region of cytokine receptors known as the ligand-binding domain, which sticks out from the cell surface. This causes the signaling domains of two receptors, which are inside the cell, to interact with one another and activate Jak-STAT signaling. Moraga et al. first tested whether ‘unnatural’ cytokine receptor pairings can trigger Jak-STAT signaling. To that end, they devised an experimental system in which the signaling domains of 10 different cytokine receptors were each fused to one of two ligand-binding domains. These ligand-binding domains were compatible with a single cytokine that, in turn, could induce any one of the 10x10=100 possible pairings of signaling domains, depending on which two were fused to the ligand-binding domains.

Moraga et al. discovered that most combinations of ligand-binding and signaling domains were able to activate Jak-STAT signaling. This confirms that the pairing of cytokine receptors is sufficient to trigger signaling inside cells and that the specificity of the interaction between cytokines and ligand-binding domains is what determines which receptors can form pairs. The researchers also showed that a cytokine receptor can even trigger signaling when forced to form a pair with a completely different class of receptor molecule, such as a receptor tyrosine kinase.

Moving beyond proof-of-principle, Moraga et al. developed a way to produce ‘unnatural’ receptor pairings in native human cells. Based on the biophysics of the interactions between cytokines and their receptors, they designed mutant versions of three cytokines that were capable of binding to only one component of their ‘natural’ receptor pairs. Two of these mutant cytokines were then linked together to create synthetic cytokines (named synthekines) that can cause one receptor for each mutant cytokine to form a pair that would not naturally occur (Figure 1).

The experiments show that Jak-STAT signaling activity downstream of two synthekines called SY1 and SY2 were distinct from those of their parent cytokines. The responses driven by synthekines generally had lower overall levels of Jak-STAT activity compared to the responses triggered by their parent cytokines. Furthermore, the patterns of STAT protein activity differed. For example, STAT1 is the most active STAT protein in the response to SY2, but least active in the response to its parent cytokines. Furthermore, a technique called high-dimensional mass cytometry revealed that other signaling pathways, particularly downstream of a protein called phosphoinositide 3-kinase, were altered. Thus, synthekines are not just a watered down combination of their parent cytokines but, instead, are new biological entities with unique signaling properties. This makes them useful research tools and promising drug candidates.

There are clear precedents for the current work (see, for example, Cunningham and Wells, 1991; Shanafelt et al., 2000; Levin et al., 2012; Junttila et al., 2012; Levin et al., 2014; Moraga et al., 2015; Ng and Galipeau, 2015). However, by presenting an expansive, multi-pronged methodology for devising and testing chimeric cytokines, Moraga et al. move the field on significantly. Furthermore, they introduce new design principles to circumvent some of the problems encountered by previous efforts; for instance, they report how to prevent the individual components of the synthekines from interacting with their ‘natural’ receptor pairs.

Of course, like all pioneering endeavors, the studies of Moraga et al. raise questions and compel further investigations. The cellular and molecular consequences of synthekines (versus their parent cytokines) have only begun to be explained, and their bioactivity in animals, particularly regarding therapeutic uses, has yet to be addressed. The latter will require the generation of synthekines that are compatible with rodents and other animal models because the current versions are made from human constituents. However, there appear to be no major obstacles to doing this.

All told, this work stands as a triumph of scientific imagination, a lucid illustration that we need not be constrained by the rules of engagement prescribed within the genome. After all, given the ever-growing practicality of synthetic biology, we are living in a time where, in the words of Lennon and McCartney, there's “nothing you can make that can't be made” (Lennon and McCartney, 1967).
